# The gut microbiome and child mental health: A population-based study

**DOI:** 10.1016/j.bbi.2022.12.006

**Published:** 2022-12-06

**Authors:** Robert Kraaij, Isabel K. Schuurmans, Djawad Radjabzadeh, Henning Tiemeier, Timothy G. Dinan, André G. Uitterlinden, Manon Hillegers, Vincent W.V. Jaddoe, Liesbeth Duijts, Henriette Moll, Fernando Rivadeneira, Carolina Medina-Gomez, Pauline W. Jansen, Charlotte A.M. Cecil

**Affiliations:** a Department of Internal Medicine, Erasmus University Medical Center, Rotterdam, The Netherlands; b Department of Epidemiology, Erasmus University Medical Center, Rotterdam, The Netherlands; c The Generation R Study Group, Erasmus University Medical Center, Rotterdam, The Netherlands; d Department of Child and Adolescent Psychiatry/Psychology, Erasmus University Medical Center, Rotterdam, The Netherlands; e Department of Social and Behavioral Sciences, Harvard. T.H. Chan School of Public Health, Boston, MA, USA; f APC Microbiome Ireland, University College Cork, Cork, Ireland; g Department of Psychiatry and Neurobehavioral Science, University College Cork, Cork, Ireland; h Department of Pediatrics, Erasmus University Medical Center, Rotterdam, The Netherlands; i Department of Pediatrics, Divisions of Respiratory Medicine and Allergology, and Neonatology, Erasmus University Medical Center Rotterdam, Rotterdam, The Netherlands

**Keywords:** Microbiome, Child mental health, Gut-brain-axis, Epidemiology, Psychiatry, Population-based

## Abstract

The link between the gut microbiome and the brain has gained increasing scientific and public interest for its potential to explain psychiatric risk. While differences in gut microbiome composition have been associated with several mental health problems, evidence to date has been largely based on animal models and human studies with modest sample sizes. In this cross-sectional study in 1,784 ten-year-old children from the multi-ethnic, population-based Generation R Study, we aimed to characterize associations of the gut microbiome with child mental health problems. Gut microbiome was assessed from stool samples using 16S rRNA sequencing. We focused on overall psychiatric symptoms as well as with specific domains of emotional and behavioral problems, assessed via the maternally rated Child Behavior Checklist. While we observed lower gut microbiome diversity in relation to higher overall and specific mental health problems, associations were not significant. Likewise, we did not identify any taxonomic feature associated with mental health problems after multiple testing correction, although suggestive findings indicated depletion of genera previously associated with psychiatric disorders, including *Hungatella*, *Anaerotruncus* and *Oscillospiraceae*. The identified compositional abundance differences were found to be similar across all mental health problems. Finally, we did not find significant enrichment for specific microbial functions in relation to mental health problems. In conclusion, based on the largest sample examined to date, we do not find clear evidence of associations between gut microbiome diversity, taxonomies or functions and mental health problems in the general pediatric population. In future, the use of longitudinal designs with repeated measurements of microbiome and psychiatric outcomes will be critical to identify whether and when associations between the gut microbiome and mental health emerge across development and into adulthood.

## 1 Introduction

The relationship between the gut microbiome and mental health has drawn immense scientific and public interest over recent years (Shoubridge et al., 2022), spurred by an increased understanding of the key role microbiome may play in mediating communication between the gut and the brain (the so-called ‘gut-brain’ axis (Cryan et al., 2019). The gut microbiome influences the brain via multiple pathways, including neurotransmitter synthesis (e.g., serotonin), activation of the immune system, production of neuroactive metabolites (e.g., short-chain fatty acids) and vagus nerve stimulation (Gershon and Margolis, 2021). Furthermore, several environmental factors that influence the brain and psychiatric risk have also been shown to impact the gut microbiome, including stress exposure, medications and diet (Cryan et al., 2019). As such, the gut microbiome has emerged as a promising potential mechanism underlying individual differences in brain function, behavior, and psychiatric risk.

To date, most studies on the gut-brain axis have been based on experimental work in animals, demonstrating the importance of gut bacteria for neurodevelopment and behavior, including learning and memory, social interactions, stress response, and anxiety- and depressive-like behaviors (Warner, 2019). A causal effect of the microbiome on the brain has further been supported by fecal microbial transplantation studies, which show that translocation of fecal bacteria from human donors with a psychiatric disorder (e.g., depression, anxiety or schizophrenia) associate with reduced microbial diversity and increased psychiatric symptoms in animals (Settanni et al., 2021). The human literature has been sparser and almost entirely comprised of clinical studies. These have focused mainly on autism spectrum disorder, implicating lower abundances of Enterococcus, Escherichia coli, Bacteroides, and Bifidobacterium in patients compared to healthy controls (Xu et al., 2019); or on major depressive disorder in adulthood, with the first large-scale, population-based studies reporting robust and independently replicated associations with lower abundance of *Prevotellaceae, Coprococcus,* and *Faecalibacterium* (Radjabzadeh et al., 2022; Sanada et al., 2020; Valles-Colomer et al., 2019). While other psychiatric symptoms have received far less attention, preliminary evidence suggests lower abundance of specific taxa in relation to disorders such attention-deficit hyperactivity disorder, schizophrenia, and generalized anxiety disorder (for a review of available evidence, see (Shoubridge et al., 2022) and (Chen et al., 2021). Besides associations with clinical disorders in adults, a smaller set of studies in infants have reported associations with subclinical mental health problems (i.e., emotional and behavioral problems) and temperamental features (e.g., (Loughman et al., 2020) and (Aatsinki et al., 2019).

Despite this rapidly expanding evidence base, key gaps remain to be addressed. First, research to date on the gut microbiome and mental health has been based on modest sample sizes (i.e., an average number of 42 cases per study (Chen et al., 2021), prone to limitations such as selection bias, unclear generalizability of findings, and low statistical power to detect associations of small effect. Second, studies have varied widely in methodology, including the use of multiple testing correction, adjustment for covariates, and the analysis of gut microbiome at different taxonomic levels, limiting comparability of findings. This has led to increased calls to move towards larger, well-designed and better powered studies that examine the gut microbiome at multiple levels (e. g., from global diversity measures to individual taxonomic units) (Shoubridge et al., 2022). Third, current studies have been based almost entirely on either infants or adults, even though more than half of psychiatric disorders emerge before the age of 18 years, and typically manifest earlier in childhood as emotional and behavioral problems (Solmi et al., 2021). As such, it remains unclear whether associations reported in adult studies are already evident during childhood. Finally, studies have focused on single psychiatric outcomes, despite evidence that psychiatric symptoms often co-occur. As such, it has not been possible to establish whether reported gut microbiome differences may be unique or shared across psychiatric symptoms.

To address these gaps, we examined cross-sectional associations between the gut microbiome and common mental health problems in a general population cohort of nearly 1,800 ten-year old children. We utilized a comprehensive approach to characterize associations between gut microbiome composition at different taxonomic levels (alpha and beta diversity measures, genus level, and functional pathway analyses) and overall psychiatric symptoms in children. As follow-up analyses, we also examined associations of the gut microbiome with eight specific domains of emotional and behavioral problems. Based on previous literature, we expected associations between gut microbiome and mental health, although we did not have any a-priori hypotheses for the direction of associations and taxa involved.

## 2 Material and methods

### 2.1 The Generation R study

The Generation R Study is a population-based prospective multi-ethnic cohort from fetal life onward conducted in the city of Rotterdam (Kooijman et al., 2016). The study was designed to identify early environmental and genetic factors and causal pathways underlying growth and development during childhood. The Generation R Study recruited 9,778 pregnant women from Rotterdam, The Netherlands, with a delivery date from April 2002 until January 2006. More than 70 % kept on participating after birth, undergoing several rounds of follow-up. Ethics approval was obtained from the Medical Ethical Committee of Erasmus MC (MEC-2012–165, October 17, 2012) and written informed consent was obtained from all participants’ parents on behalf of their children. All methods were performed in accordance with the Declaration of Helsinki. In total, 5,862 children participated in the wave at age 10 years, of which 2,526 children returned a stool sample that could be included in the microbiome dataset (Radjabzadeh et al., 2020) and 5,523 mothers returned a valid Child Behavior Checklist (CBCL) (Kooijman et al., 2016). Of these, we excluded participants with no data on microbiome and CBCL, whose technical covariates were not available, whose stool sample was in mail for more than 5 days, and who did not genetic data available. A final sample of 1,784 participants remained, and were thus included in the present cross-sectional observational study (sample filtering in Supplementary Table 1, appendix A). The study is outlined and reported following the guidelines for human microbiome research (checklist in Supplementary, appendix B) (Mirzayi et al., 2021).

### 2.2 Gut microbiome

#### 2.2.1 Stool sample selection

Stool (i.e., feces) samples were collected at a mean age of 9.8 years (SD = 0.3), as described in more detail elsewhere (Radjabzadeh et al., 2020). In brief, samples were collected at home by the participants using a Commode Specimen Collection System (Covidien, Mansfield, MA). An aliquot of approximately 1 g was transferred to a 25 × 76 mm feces collection tube (Minigrip Nederland, Lelystad, The Netherlands) without preserving agent included and sent through regular mail to the Erasmus MC. In case of delay (i.e., defecation in evening or weekend), samples were asked to be stored by participants at 4 ^◦^C (home fridge) before mailing to Erasmus MC. A short questionnaire addressing date and time of defecation and current or recent use of antibiotics (past year) was filled out by the primary caregiver and included in the package. Upon arrival at Erasmus MC, samples were recorded and stored at −20 °C.

#### 2.2.2 DNA isolation and sequencing

DNA isolation, 16S rRNA profiling and filtering were performed as previously described (Radjabzadeh et al., 2020). In brief, stool samples were randomly taken from the −20 °C freezer and allowed to thaw for 10 min at room temperature prior to DNA isolation. DNA was isolated using the automated Arrow Stool DNA isolation kit (Isogen Life Science, De Meern, The Netherlands) after bead beating with 0.1 mm silica beads (MP Biomedicals, LLC, Bio Connect Life Sciences BV, Huissen, The Netherlands). The V3 and V4 variable regions of the 16S rRNA gene were amplified using the 319F-806R primer pair and dual indexing (Fadrosh et al., 2014) followed by Illumina MiSeq sequencing (Illumina Inc., San Diego, CA) on the V3 flowcell (MiSeq Reagent Kit v3, 2 × 300 bp) at an average depth of 50,000 read-pairs per sample. Raw reads from Illumina MiSeq were demultiplexed using a custom script to separate sample fastq files based on the dual index. Primers, barcodes and heterogeneity spacers were trimmed off using tagcleaner v0.16 (Schmieder et al., 2010). Trimmed fastq files were loaded into R v4.0.0 (R Core Team, 2020) with the DADA2 (Callahan et al., 2016) package. Quality filtering was performed in DADA2 using the following criteria: trim = 0, maxEE = c(2,2), truncQ = 2, rm.phix = TRUE. Filtered reads were run through the DADA2 ASV assignment tool to denoise, cluster and merge the reads. ASVs were assigned a taxonomy from the SILVA version 138.1 rRNA database (Quast et al., 2012) using the RDP naïve Bayesian classifier (Wang et al., 2007). The resulting data tables were combined into a phyloseq object using Phyloseq (McMurdie and Holmes, 2013) and a phylogenetic tree was generated using the Phangorn R package (Schliep et al., 2016) based on the sequences of the ASVs and added to the phyloseq object. PICRUSt2 prediction of functional MetaCyc microbial pathways was performed in R using PICRUSt2 (Douglas et al., 2020) with the default EPA-NG (Barbera et al., 2019) placement option and MinPath (Ye and Doak, 2009) biological pathway reconstruction based on Enzyme Commission (EC) numbers (Caspi et al., 2018).

### 2.3 Child mental health problems

Child mental health problems were assessed using the Child Behavior Checklist 6–18 (CBCL/6–18) at age 10 rated by mothers. The mothers rated various emotional (i.e., internalizing) and behavioral (i.e., externalizing) problems of the child in the previous six months using 118 questions on a three-point scale (0 = not true, 1 = somewhat true, 2 = very true). Based on these items, three scales measuring overall psychiatric symptoms (i.e., internalizing problems, externalizing problems, and total problems) were derived, as well as eight specific domains of emotional and behavioral problems (i.e., anxious/depressed, withdrawn/depressed, somatic complaints, social problems, thought problems, attention problems, rule-breaking behavior and aggressive behavior) (Achenbach and Rescorla, 2001).

### 2.4 Other variables

Based on previous work in our cohort validating the microbiome data, we included age, sex, body mass index (BMI), use of antibiotics, technical covariates, and genetic principal components as covariates (for more details on technical covariates, see (Radjabzadeh et al., 2020). Age was recorded as the date of stool sample production. Sex was obtained through midwives at birth (Kooijman et al., 2016). BMI, which has been found to strongly associate to microbiome composition (Radjabzadeh et al., 2020) and partly reflects aspects of diet (Huang et al., 2004), such as energy intake, was calculated from weight and length recorded during a research visit when children were 10 years old. Use of antibiotics was measured at the time of stool collection using self-report, and was categorized into three categories (i.e., last month, 1 to 3 months ago, 3 months to a year ago). Five technical covariates were included in all analyses, being i) time in mail (i.e., the number of days between stool sample production and arrival of the sample in Erasmus MC, max. 5 days); ii) season of stool sample production (winter, spring, summer, fall); iii) one of two DNA isolation batches that were observed during dataset generation; iv) one of three sequencing run batches; and v) the number of sequencing reads. Genetic principal components (PCs) were derived from DNA, in order to adjust for population stratification (first 10 PCs included in the analyses), included in light of the genetic contribution dietary variation (Cole et al., 2020) and the highly heterogeneous and the admixed nature of our sample (Medina-Gomez et al., 2015). More detail on how genetic PCs were derived from DNA can be found in (Medina-Gomez et al., 2015), which used a similar approach. In brief, genotyped data was merged, after which around 27,000 independent high quality SNPs (MAF > 0.05) from the 1000 Genomes phase 3 (Genomes Project C, 2015) were selected. Based on this pruned dataset, pairwise identity-by-state relations were calculated for each pair of individuals using PLINK (Purcell et al., 2007). Multi-dimensional scaling was subsequently used to derive genetic PCs from this matrix. Further, maternal education at child birth was included as a proxy of socioeconomic status, an important covariate in microbiome research applied to mental health (Zhu et al., 2020). Maternal education was assessed by a questionnaire (Kooijman et al., 2016) and defined by the highest attained educational level and classified into six categories (i.e., no education; primary education; lower vocational training or intermediate general school; >3 years secondary school or intermediate vocational training or higher vocational training; bachelor’s degree; higher academic education or PhD). Finally, country of origin of the participant was used to describe the study sample. Country of origin was determined based on self-reported country of birth of four grandparents (Kooijman et al., 2016). A participant was deemed to be of non-Dutch origin if one parent was born abroad. If both parents were born abroad, maternal country of birth was leading. The following categories were recorded (Kooijman et al., 2016): Dutch, Indonesian, Cape Verdean, Moroccan, Dutch Antilles, Turkish, Surinamese-Creole, Surinamese-Hindustani, Surinamese-unspecified, and Japanese. Children with a country of origin other than these were grouped as: African, North American, Central/South American, Asian, European, or Oceanian.

### 2.5 Analyses

Analyses were performed in R version 4.1.0 (R-Core-Team, 2016) with Bioconductor 3.13 (Huber et al., 2015) adjusting for all covariates as specified in the previous section. The only covariate with missing data (maternal education) was imputed by multiple imputation using chained equations with the “mice” package in R (Van Buuren and Groothuis-Oudshoorn, 2011). 30 datasets were generated using 100 iterations. Pooled estimates were obtained using Rubin’s rules (Rubin, 1987), and if pooling functions were not available, median statistics were reported. As microbiome data is compositional and zero-inflated, zero abundances were imputed using the zCompositions package (Palarea-Albaladejo and Martín-Fernández, 2015) and transformed by centered log ratio transformation. Child mental health problems were square root transformed to approach normality.

Analyses were performed using a hierarchical approach. For our primary aim, we assessed associations with overall psychiatric symptoms (i.e., internalizing problems, externalizing problems, and total problems); as follow-up analyses, we examined associations with individual empirical syndrome scales (i.e., anxious/depressed, withdrawn/depressed, somatic complaints, social problems, thought problems, attention problems, rule-breaking behavior and aggressive behavior). All scales present continuous variables and were modelled dimensionally.

Analyses proceeded in three steps focusing on different taxonomic levels, as described below. All corresponding scripts are available in the Supplementary, appendix C.

#### 2.5.1 Step 1. Associations with alpha diversity indices

Associations of alpha diversities (i.e., microbiome richness (Fisher et al., 1943), Shannon diversity (Shannon, 1948), and inverse Simpson index (Simpson, 1949) with square root transformed child mental health problems were examined using linear regression models. Alpha diversity indices are one-value summaries of a sample’s microbiome profile that reflect the number of observed species and their mutual distribution. At this step, we additionally performed a sensitivity analysis, repeating analyses in a subsample of participants whose time in mail (of stool sample) was 3 days or less (as opposed to 5 days max.) to ascertain that results were robust when using a more stringent time window.

#### 2.5.2 Step 2. Associations with gut microbiome composition (genus-level)

Univariate (i.e., per taxon) differential abundance analyses and multivariate (i.e., including all taxa in the profile) analyses were performed on the genus-level abundance table. Abundance data were transformed to centered log ratios after imputing zero read abundances to reduce biases introduced by the compositional nature of the microbiome data.

Univariate associations of single taxa with child mental health problems were determined by ANCOM-BC differential abundance analysis (Lin and Peddada, 2020) using the square root transformed child mental health problems. Univariate taxon analyses determine the associations with single taxa without considering the entire microbiome profile and mutual relations between taxa. Of note, number of sequencing reads was not included as a covariate in this analysis, as ANCOM-BC runs analyses by first estimating sampling fractions. Further, as differential abundance methods often produce different results, we additionally ran linear regression models to triangulate findings.

Multivariate composition was associated with child mental health problems by PERMANOVA (“adonis” function in R library vegan (Oksanen et al., 2013) using the pairwise Euclidean distance matrix of the centered log ratio profiles (beta diversity), using 999 permutations, and adding the square root transformed phenotype as last variable into the base model. PERMANOVA estimates whether the microbiome profiles of samples within a category are more similar (cluster together) than between categories using the entire profile to determine dissimilarities between samples.

#### 2.5.3 Step 3. Functional pathways

MetaCyc relative functional pathway abundances (Caspi et al., 2014) were associated using the base model and linear regression with the square root of child mental health problems as outcomes. Based on the taxa present in each microbiome profile, PICRUSt predicts the level of enzymes that are present in each sample using databases, MetaCyc pathway abundances are then derived from these predicted enzyme levels (Caspi et al., 2014).

#### 2.5.4 Multiple testing

Multiple testing adjustment was performed using Benjamini-Hochberg correction (Benjamini and Hochberg, 1995) within each analyses step and for each outcome individually, presented as *p*-corrected. Results were considered significant for *p*-corrected < 0.05 and nominally significant for *p* < 0.01 and *p*-corrected > 0.05.

## 3 Results

**Fig. 1:**
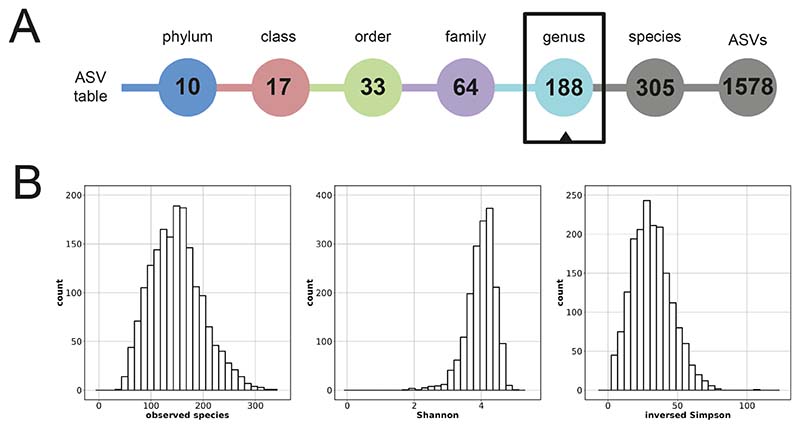
Microbiome characteristics. Characteristics of the stool microbiome datasets used in this study based on all 1,784 samples. (A) Overview of the number of taxa at different taxonomic levels. (B) Distributions of alpha diversities.

Sample characteristics are presented in Table 1. Mean age of the participants was 9.8 years (SD = 0.3 years) during stool sample production and 9.7 years (SD = 0.3 years) during child mental health problems measurement. Mean time-lag between stool sample and mental health measure was 0.1 years (SD = 0.2). Mean BMI was 17.2 kg/m^2^. A total of 16 ethnicities were recorded with Dutch (66.3 %), non-Dutch European (7.5 %), Turkish (3.7 %) and Moroccan (3.4 %) being the most common. Microbiome characteristics are displayed in Fig. 1 and Supplementary Table 2, appendix A. A total of 305 different species were detected in the dataset after QC and filtering. Each sample contained on average 84.2 different species. At genus-level, 188 different genera were detected; and on average 61.1 genera were present in each sample. Descriptives of child mental health measures are displayed in Supplementary Table 3, appendix A. Correlations between measures of microbiome composition, mental health outcomes, and covariates were small to moderate (Supplementary Fig. 1, appendix A).

### 3.1 Child mental health problems and gut microbiome diversities

After adjusting for age, sex, BMI, self-reported use of antibiotics, maternal education, technical covariates, and the first 10 genetic PCs, we did not find any associations between overall mental health problems and either gut microbiome richness or the two diversity indices in children (Table 2). Most associations were negative in direction (i.e., greater mental health problems relating to lower microbiome diversity), but not significant at either an adjusted or nominal threshold (see for scatterplots Supplementary Fig. 2, appendix A). Follow-up analyses examining specific mental health problems (Table 2), as well as sensitivity analyses focusing on a subsample with stricter time-in-mail exclusion criteria (Supplementary Table 4, appendix A), showed similar findings.

### 3.2 Child mental health problems and gut microbiome profiles

**Fig. 2:**
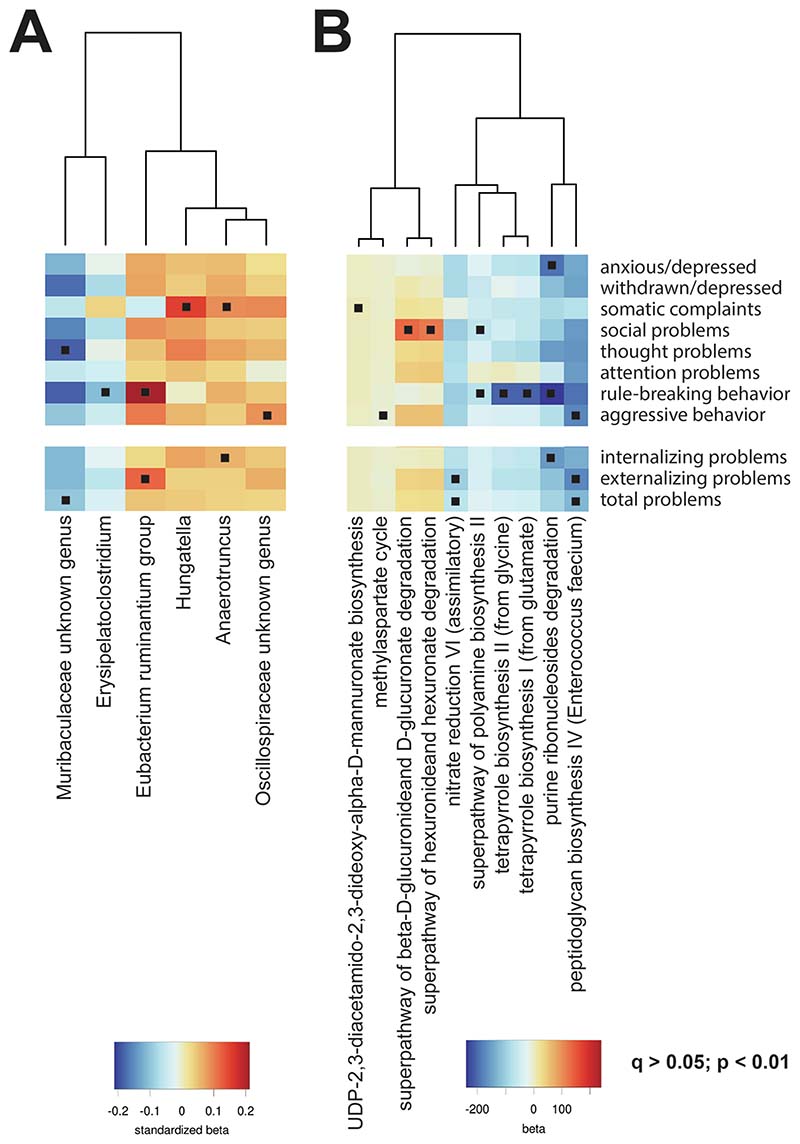
Associations of gut microbiome genera and functional pathways with mental health problems. (A) Genus-level taxonomies that were nominally significant (*p* < 0.01 and *p*-corrected > 0.05; indicated by a square) associated with one of the mental health problems after covariate adjustment are listed in the heatmap. Color represents the standardized beta from the ANCOMBC function. (B) Predicted microbial functions organized in MetaCyc pathways that were nominally significant (*p* < 0.01 and *p*-corrected > 0.05; indicated by a dot) significant associated with one of the mental health problems after covariate adjustment are listed in the heatmap. Colors represent the standardized beta obtained from the LM linear model function.

We analyzed single taxonomies associated with child mental health problems using univariate ANCOM-BC differential abundance models. We identified a total of 6 genera nominally associated with either overall or specific mental health problems, based on *p* < 0.01 (i.e., *Muribaculaceae unknown genus*, *Erysipelatoclostridium*, *Eubacterium ruminantium group*, *Hungatella*, *Anaerotruncus,* and *Oscillospiraceae unknown genus*; for full results, see Supplementary Table 1-11, appendix D). These associations, however, did not survive correction for multiple testing. Of note, there was little evidence of symptom specificity, with the six genera associating with mental health phenotypes in the same direction (Fig. 2A). Univariate models based on linear regressions produced similar results (Supplementary Table 1-11, appendix E).

A multivariate approach was performed to investigate if overall gut microbiome compositions are associated with mental health problems in children. A PERMANOVA test was performed on the pair-wise dissimilarities between samples to detect clustering of microbiome profiles associated with overall and specific child mental health problems, while controlling for covariates. No significant associations were detected at either adjusted or nominal significance levels (Supplementary Table 5, appendix A). See for ordination plots Supplementary Fig. 3, appendix A.

### 3.3 Child mental health problems and gut microbial functions

The functional content of gut microbiome was predicted from the ASV-level abundance tables using the PICRUSt2 tool and combined in annotated MetaCyc pathways (Supplementary Table 1-11, appendix F), and run as univariate models with linear regression while adjusting for covariates. Nominally significant associations were found for 10 pathways across overall and specific mental health problems (Fig. 2B). As with other findings, these associations did not survive multiple testing correction.

## 4 Discussion

We examined associations between gut microbiome and child mental health problems at different taxonomic levels (alpha and beta diversity measures, genus-level, and functional pathways), drawing on data from a large population-based study of almost 1,800 10-year-old children. Overall, we found no clear evidence of a cross-sectional association between gut microbiome composition and either overall or specific child mental health problems. The direction and magnitude of associations for each taxon was similar across mental health outcomes, reflecting the correlated nature of emotional and behavioral problems. Furthermore, although some of the identified nominal associations were consistent with previous findings, implicating genera such as *Hungatella*, *Anaerotruncus* and *Oscillospiraceae*, none of these associations survived multiple-testing correction. We also did not identify any enriched microbial functional pathways in relation to child mental health problems. Together, our findings do not support a strong link between the gut microbiome and mental health in the general pediatric population at this age.

We first examined the relationship between indices of gut microbial diversity and child mental health problems. We found that although generally lower gut microbial diversity and richness was linked to more overall (e.g., internalizing problems) as well as specific (e.g., anxious/depressed behavior) mental health problems, these associations were not significant. While reduced microbial diversity has been robustly associated with variables such as older age, higher BMI and use of antibiotic, the relationship with mental health problems other than adult depression and autism spectrum disorder has been inconsistent (Xu et al., 2019; Sanada et al., 2020; Chen et al., 2021). These findings are in line with other population-based studies on the association between gut microbiome and mental health, which as well reported weak associations with alpha diversity indices in children and adults (Loughman et al., 2020; Radjabzadeh et al., 2022; Valles-Colomer et al., 2019). As a second step, we found that no single genus was associated with overall or specific mental health problems after multiple-testing correction. Microbiota that best explained mental health problems in ANCOM-BC overlapped with those identified using linear regression models as an alternative analytical method. Associations were generally found to be non-specific, with effects being of similar magnitude and direction across phenotypes. This is perhaps unsurprising given the known co-occurrence between mental health problems, and highlights the importance of assessing multiple psychiatric outcomes concurrently. Such an approach, however, contrasts with what is most commonly done in the field, where individual psychiatric outcomes are typically examined in isolation (Chen et al., 2021). Indeed, one study reported associations with gut microbiome and having elevated symptoms in at least one domain of overall mental health problems (Loughman et al., 2020), although associations with specific domains were not examined, thereby precluding comparisons across phenotypes. Similarly to the genus and single taxon analyses, results from the multivariate PERMANOVA were not significant after multiple-testing correction. Overall, the small effect sizes observed in our study are in line with what has been reported by other population *omics* research on mental health, including those of genetic, epigenetic, and transcriptomic nature (Xu et al., 2019; Sanada et al., 2020; Chen et al., 2021).

While no associations survived multiple testing correction, we would like to highlight three genera that were most consistently associated with child mental health problems across analyses at a nominal level (i. e., *p* < 0.01, but *p*-corrected > 0.05), and that have been associated with psychiatric phenotypes in previous literature. First, we found that a one standard deviation higher abundance of *Hungatella* was nominally associated with a 0.15 standard deviation (95 % confidence interval [0.05, 0.26]) increase in somatic complaints, a feature of internalizing problems. Interestingly, increased abundance of *Hungatella* has been previously found to associate with infant temperament (Wang et al., 2020), and notably also to depression in a large population-based sample of adults – a finding that was also replicated in an independent cohort (Radjabzadeh et al., 2022). Together, these findings point to *Hungatella* as an interesting candidate for future research, particularly in relation to features of depression and its developmental precursors in childhood. Further, one standard deviation higher abundance of the *Anaerotruncus* genus was nominally associated with 0.06 to 0.09 standard deviation (95 % confidence interval [0.02, 0.11] and [0.02, 0.16]) more internalizing problems and somatic complaints, in accordance with broader literature. Although only consisting of small individual studies, increased *Anaerotruncus* has been implicated in several psychiatric diagnoses, such as schizophrenia and anorexia nervosa (Nikolova et al., 2021), but also nominally in major depressive disorder (Zhang et al., 2021:). Finally, we found that one standard deviation higher abundance of *Oscillospiraceae* was nominally associated with 0.09 standard deviation (95 % confidence interval [0.02, 0.15]) more child aggressive problems*.* This is in contrast to what has been observed previously for other outcomes, with a systematic review reporting *lower* abundance of this genus in relation to major depressive disorder in adults (Barandouzi et al., 2020), as well as cholesterol and obesity in childhood (Maya-Lucas et al., 2019), although higher abundance of *Oscillospiraceae* has been identified in association to child sleep apnea syndrome (Valentini et al., 2020).

As a final step, we performed a functional analysis to examine whether specific microbial functions may be enriched in relation to child mental health problems. We did not find any functional pathways to be enriched after multiple-testing correction for overall mental health problems. With regards to nominally enriched pathways (i.e., *p* < 0.01, but *p*-corrected > 0.05), we found that microbiome profiles associated with aggressive behavior were enriched for the methylaspartate cycle. Multiple lines of evidence support a role of the *N*-methyl-D-aspartate receptor in the pathophysiology of several psychiatric disorders such as schizophrenia (Weickert et al., 2013) and bipolar disorder (Mundo et al., 2003), although exact mechanisms remain unclear. Further, internalizing behavior, anxiety, and rule-breaking behavior all showed negative enrichment for a pathway implicated in purine metabolism (i.e., purine ribonucleosides degradation). Previous work has indicated that metabolites involved in purine metabolism are down-regulated in children and adolescents with major depressive disorder as compared to healthy controls (Zhou et al., 2019). Relatedly, depressive symptoms in adolescents have been found to associate with increased serum uric acid (Tao and Li, 2015) – the end-product of the purine metabolism – which acts as a compensatory mechanism against increased oxidative stress observed in depression (Kang and Ha, 2014; Bhatt et al., 2020). While these suggestive findings point to potential metabolic functions that may be implicated in child mental health at the level of the gut microbiome, it is not known which specific enzymes in the pathway account for the observed enrichment. As such, molecular studies will be needed in future to establish the robustness of findings and their potential functional relevance.

Overall, we do not find evidence for a clear link between the gut microbiome and mental health problems in children. This contrasts with the existing literature, which has been rife with reports of significant associations – although few consistent findings have emerged based on recent systematic reviews (Xu et al., 2019; Sanada et al., 2020; Chen et al., 2021). Weak to no associations may be explained by several factors. First, we examined mental health problems dimensionally in the general population, as opposed to most previous research performing case-control comparisons in patient samples. Dimensional analyses of continuous variables have the advantage of modelling the full spectrum of symptoms as a continuum in the general population. However, if associations with the gut microbiome manifest only at more severe ends of the symptom spectrum, our study may have lacked the symptom severity necessary to detect such associations. Second, we investigated common mental health problems in children, which precede the peak onset of several psychiatric disorders that have been linked to the gut microbiome in adults, such as major depression or schizophrenia. Here, we do not find evidence that the gut microbiome associates significantly with developmental precursors for these disorders (e.g., emotional and thought problems) in childhood. On the one hand, it is possible that associations with the gut microbiome only emerge during specific developmental periods, for instance once a psychiatric disorder is fully manifested. On the other hand, it is also possible that differences in composition observed in adults may be more likely a consequence rather than a risk factor for these psychiatric disorders, for example because of medication use, dietary and lifestyle changes associated with the disorder, as has been observed for autism spectrum disorder (Yap et al., 2021). Finally, a possible interpretation of our results is that the gut microbiome does not substantially affect mental health problems, and that previously reported associations may have been biased by factors such as unmeasured confounding, small sample sizes and inadequate adjustment for false positives.

### 4.1 Limitations and future directions

Our findings should be interpreted considering several limitations. First, similarly to other population-based studies in children and adults (Loughman et al., 2020; Radjabzadeh et al., 2022; Valles-Colomer et al., 2019), gut microbiome data was processed using 16S rRNA sequencing instead of using the more precise whole genome sequencing (Laudadio et al., 2018), due to the prohibitive cost of implementing this approach at large scale. To mitigate potential drawbacks of 16S rRNA sequencing, we used DADA2 for denoising the data and PICRUSt2 for pathway predictions. These tools produce more similar results for 16S rRNA and whole genome sequencing (Langille et al., 2013) than previous 16S rRNA pipelines. Moreover, 16S rRNA sequencing is currently the most widely used application to study microbiota, making our study comparable with others in the field. Future efforts should be placed on (partly) triangulating different sequencing methods in the same study, to increase robustness of findings. Second, although we included a range of covariates in our analyses, it was not possible to account for other potentially important variables such as dietary patterns, medication use and intrapartum antibiotic prophylaxis (although we included recent use of antibiotics during the past year as a covariate). Yet, the use of (psychiatric) medication in this pediatric population, particularly at this age, is expected to be low compared to patient samples. Third, gut microbiome was estimated from stool samples; however, we do not know how well stool samples reflect the microbiome of the full gastrointestinal tract. Also, we collected stool samples at room temperature, which can affect survival of anaerobes (Radjabzadeh et al., 2020).

Our findings also point to key avenues for future research. First, while the intent of this study was to investigate associations with common mental health problems in children, it would also be valuable to examine associations with autism spectrum disorder (ASD) in the general pediatric population, which is currently the most examined child brain-based disorder in relation to the gut microbiome (Xu et al., 2019). Second, our study, like many others in the field, was cross-sectional. An important step for future research will be the assessment of longitudinal data at repeated time points, to clarify the direction of associations between the gut microbiome and mental health, and to test whether associations emerge during specific developmental periods. Of note, one study that did assess gut microbiome repeatedly (at age 1, 6 and 12 months) and associated this to mental health at 2 years established only proximal associations (i.e., with gut microbiome at age 12 months, and not for microbiome assessed at age 1 and 6 months) (Loughman et al., 2020). Third, future studies can also investigate markers of physiological disease as outcomes, and thereby assess the role of discrete measures of functional outputs (e.g., short chain fatty acids, pH levels or markers for systemic immune dysregulation) in the association between microbiome and psychiatric risk. Further, it will be important to characterize how the gut microbiome associates with individual differences within the brain *in vivo* during development, for example through the use of structural and functional neuroimaging. Fourth, with data on almost 1,800 children, our study is substantially larger than published research (Xu et al., 2019; Sanada et al., 2020; Chen et al., 2021). Yet, it is still possible that associations between the gut microbiome and child mental health problems in the general population may be too subtle to be detected with our current sample alone. Consequently, as more pediatric population-based cohorts with gut microbiome and mental health data during childhood become available, it will be important in future to pool results via meta-analysis in order to further maximize power and detect potentially subtle but robust associations.

### 4.2 Conclusion

Based on this large, population-based study, we find little evidence of an association between the gut microbiome and common mental health problems in children. Our study does not definitively refute a link between the gut microbiome and child mental health problems but indicates that associations are likely of small magnitude in the general pediatric population at this age. In future, collaborative initiatives pooling results from multiple studies will be needed to maximize statistical power to detect subtle associations. Furthermore, the use of longitudinal data from early life to adulthood will mark a crucial step for understanding the role of the gut microbiome in the development of psychiatric symptoms and characterizing how associations between these factors unfold over time.

## Supplementary Material

Supplementary Material

## Figures and Tables

**Table 1:** Baseline characteristics of the total cohort participating in the age 10 years data collection wave compared to the sub-cohort with child mental health problems and microbiome data.

	CBCL at age10 and microbiome	Participated in CBCL at age 10	*P*
*N*	1,784	5,523	
Age, years (mean (SD))	9.8 (0.29)	9.7 (0.33)	<0.001
Sex, girls (%)	48.8	50.1	0.342
BMI, kg/m^2^ (mean (SD))	17.2 (2.43)	17.4 (2.55)	0.012
Use of antibiotics, yes (%)	7.3		
Last month (%)	1.2		
1–3 months ago (%)	2.0		
3 months to 1 year ago (%)	4.1		
Education level mother at child birth			0.069
No education (%)	0.1		
Primary education (%)	4.3		
Lower vocational training, intermediate general school (%)	8.2		
>3 years secondary school or vocational training	27.9		
Bachelor’s degree	25.4		
Higher academic education or PhD	29.1		
Missing (%)	5.0	6.0	
Country of origin			0.208
Dutch (%)	66.3	64.4	
Non-Dutch (%)	32.9	34.4	
Missing (%)	0.8	1.2	

Continuous data was compared using t-statistics, while categorical data was compared using Chi-square statistics. CBCL: Child Behavior Checklist, the child mental health problems; SD: standard deviation.

**Table 2:** Associations between child mental health problems and alpha diversities.

	Microbiome richness			–	Shannon diversity			–	Inversed Simpson diversity		
	B	se	*p*		B	se	*p*		B	se	*p*
Overall psychiatric symptoms											
Internalizing problems	–0.941	0.665	0.157		–0.012	0.008	0.143		–0.530	0.288	0.066
Externalizing problems	0.185	0.636	0.771		0.001	0.008	0.884		0.115	0.276	0.676
Total problems	–0.182	0.441	0.680		–0.005	0.006	0.369		–0.177	0.191	0.355
Specific domains of emotional and behavioral problems											
Anxious/depressed	–1.361	0.803	0.091		–0.015	0.010	0.134		–0.668	0.349	0.055
Withdrawn/depressed	–0.053	0.962	0.956		–0.005	0.012	0.696		–0.062	0.418	0.881
Somatic complaints	–0.525	0.923	0.570		–0.007	0.012	0.532		–0.418	0.401	0.297
Social problems	–0.276	0.872	0.752		–0.014	0.011	0.208		–0.513	0.379	0.176
Thought problems	–0.107	0.888	0.904		–0.009	0.011	0.439		–0.369	0.385	0.338
Attention problems	0.495	0.776	0.523		0.004	0.010	0.706		0.261	0.337	0.438
Rule-breaking behavior	0.211	1.043	0.840		0.001	0.013	0.915		0.047	0.453	0.918
Aggressive behavior	0.116	0.694	0.867		0.001	0.009	0.872		0.133	0.301	0.658

Linear regression of child mental health problems and gut microbiome alpha diversities. Model: alpha diversity measure ˜ sqrt(child mental health phenotype) + age + sex + BMI + self-reported use of antibiotics + maternal education + time in mail + season of stool production + DNA isolation batch + sequencing run batch + number of sequencing reads + first 10 genetic PCs. Values are pooled from 30 imputed datasets. Alpha: alpha diversity metric; sqrt: square root transformed; PCs: principal components; se: standard error; *p*: *p*-value before correction.

## Data Availability

Data will be made available on request.
