# Supplementary material for: The gut microbiome and child mental health: A population-based study
Source: Brain Behav Immun. Author manuscript; Available in PMC 2023 Feb 9. (PMC7614161; doi:10.1016/j.bbi.2022.12.006)
Supplement: Supplementary Material [file EMS164585-supplement-Supplementary_Material.zip › 1-s2.0-S0889159122004640-mmc1.docx]

**SUPPLEMENTARY METHODS, TABLES AND FIGURES**

**The gut microbiome and child mental health: a population-based study**

Robert Kraaij^1^*****, Isabel K. Schuurmans^2,3^*****, Djawad Radjabzadeh^1^, Henning Tiemeier^4,5^, Timothy G. Dinan^6,7^, André G. Uitterlinden^1,2^, Manon Hillegers^4^, Vincent W.V. Jaddoe^8^, Liesbeth Duijts ^8,9^, Henriette Moll^8^, Fernando Rivadeneira^1^, Carolina Medina-Gomez^1^, Pauline W. Jansen^4^, Charlotte A.M. Cecil^4,2^

*****These authors contributed equally

^1^Department of Internal Medicine, Erasmus University Medical Center, Rotterdam, The Netherlands

^2^Department of Epidemiology, Erasmus University Medical Center, Rotterdam, The Netherlands

^3^The Generation R Study Group, Erasmus University Medical Center, Rotterdam, The Netherlands

^4^Department of Child and Adolescent Psychiatry / Psychology, Erasmus University Medical Center, Rotterdam, The Netherlands

^5^Department of Social and Behavioral Sciences, Harvard. T.H. Chan School of Public Health, Boston, MA, USA

^6^APC Microbiome Ireland, University College Cork, Cork, Ireland.

^7^Department of Psychiatry and Neurobehavioral Science, University College Cork, Cork, Ireland.

^8^Department of Pediatrics, Erasmus University Medical Center, Rotterdam, The Netherlands

^9^Department of Pediatrics, divisions of Respiratory Medicine and Allergology, and Neonatology, Erasmus University Medical Center Rotterdam, Rotterdam, the Netherlands

**
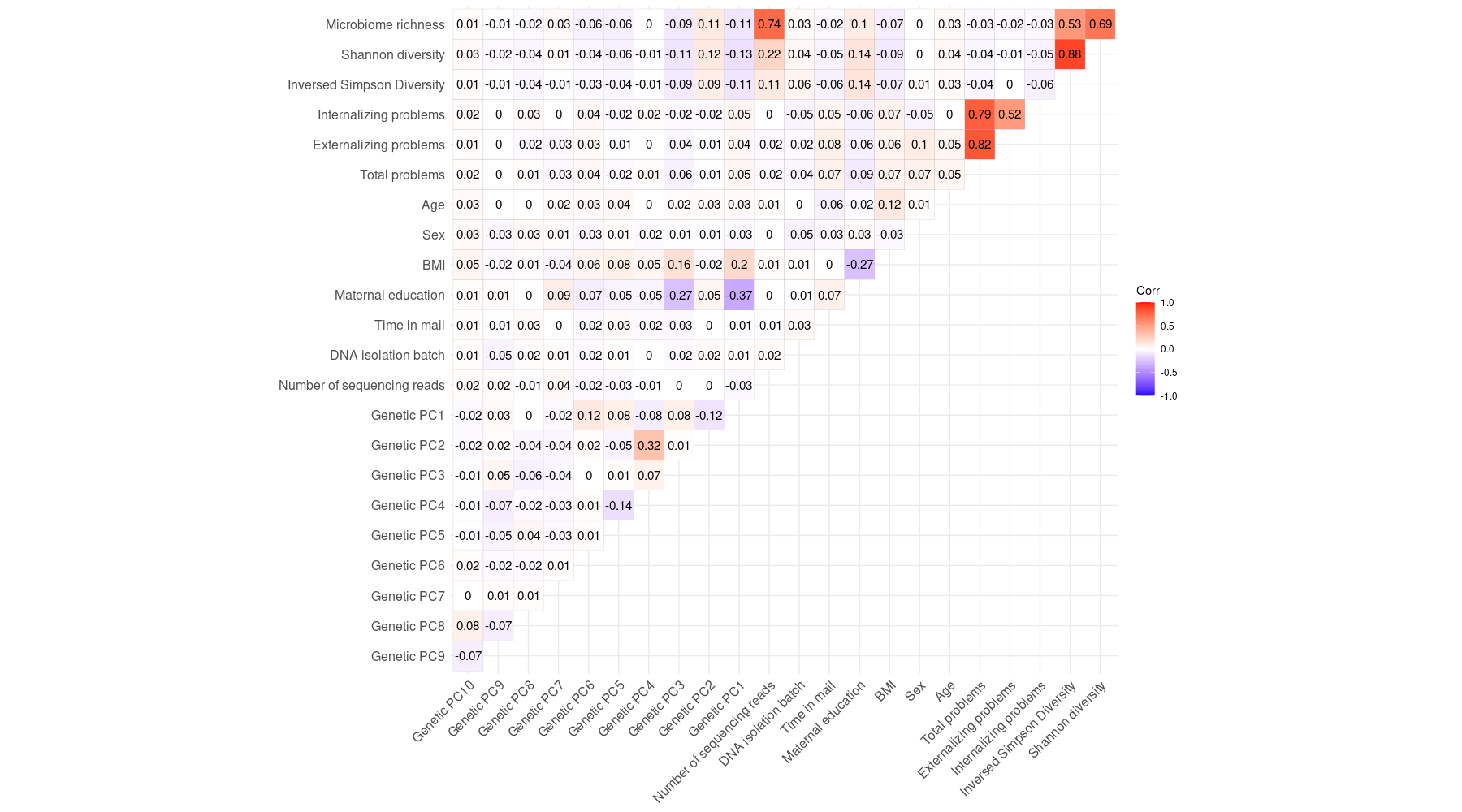
Supplementary Figure 1. Correlation table for study variables**

CBCL scales (internalizing problems, externalizing problems, total problems) are square root transformed to approach normality. Correlations for antibiotics use, season and sequencing batch were not included as these are categorical variables with >2 levels. Pearson correlations are shown for all variables, and point-biserial correlations for sex and DNA isolation batch


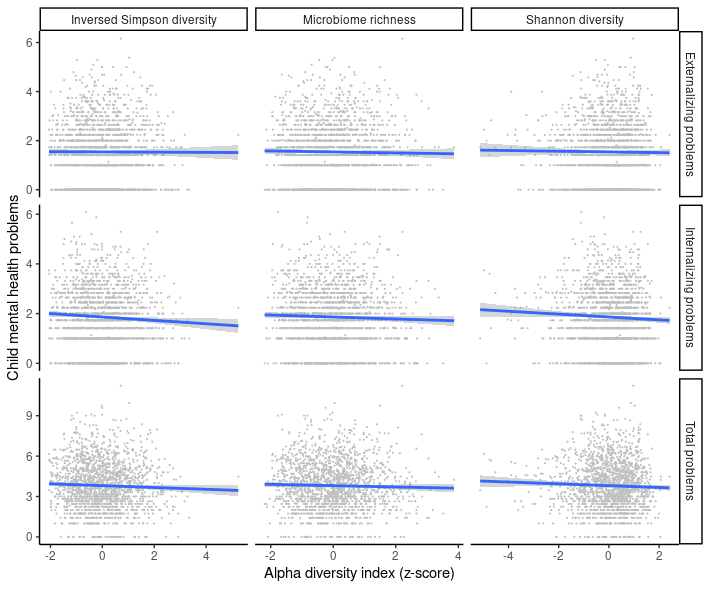
**Supplementary Figure 2. Scatterplot showing correlation between alpha diversity indices and overall psychiatric symptoms**

CBCL scales (internalizing problems, externalizing problems, total problems) are square root transformed to approach normality. Alpha diversity indices were scaled.

**Supplementary Figure 3. Ordination plots PERMANOVA**

**
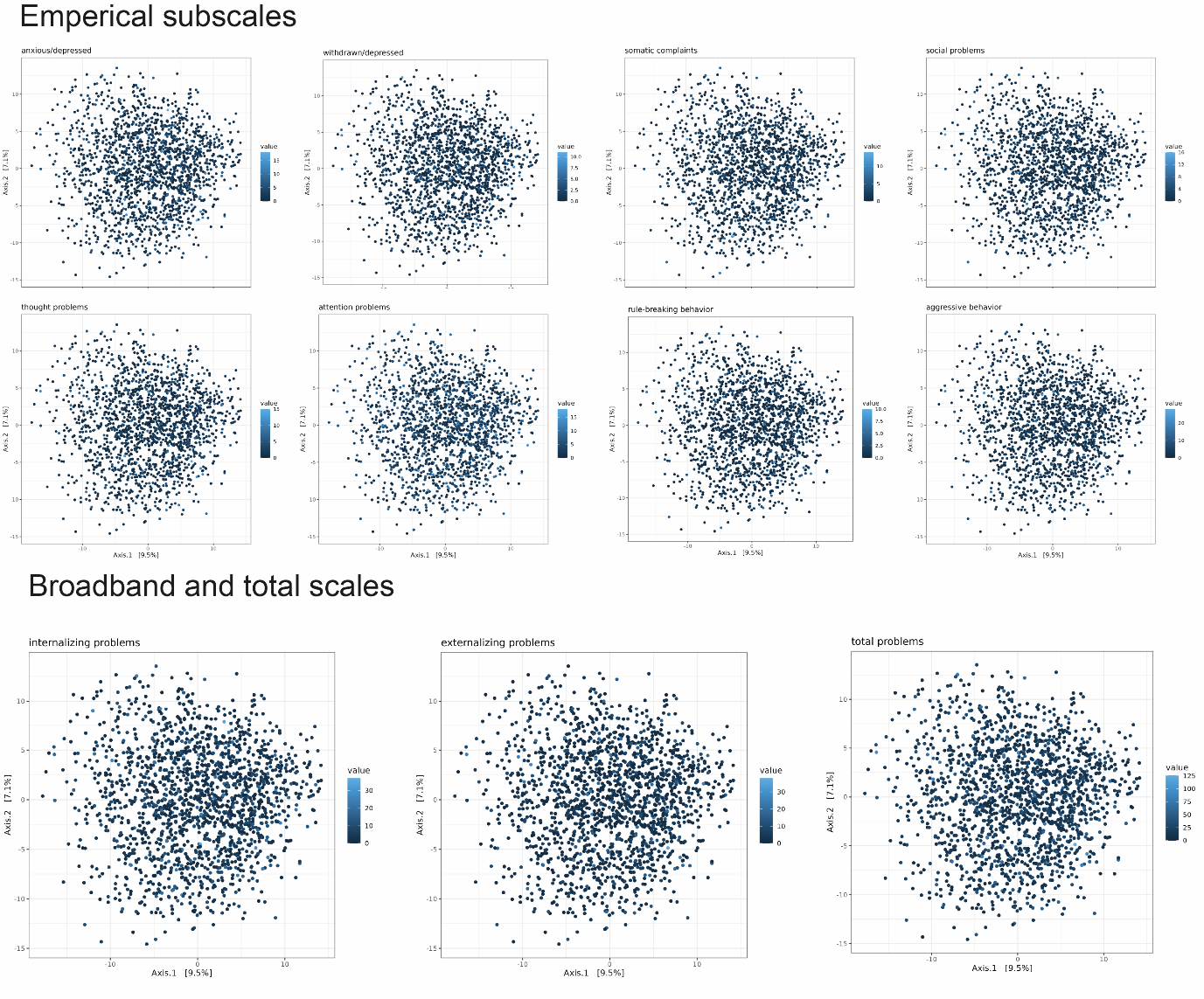
**

Ordination plots of CBCL scales and first to principal coordinates. Coloring is based on CBCL score as indicated at the right of each plot.

**Supplementary Table 1. Sample filtering**

| Filter | *N* |
| --- | --- |
| None (wave at age 9 participants) | 2,526 |
| Technical covariates available | 2,275 |
| Not exceeding time in mail (days) > 5 | 2,187 |
| Child mental health problems available | 1,948 |
| Genetic PCs / consent available | 1,784 |

PCs: principal components

**Supplementary Table 2. Microbiome characteristics**

|  | | % | mean (SD) | *N* |
| --- | --- | --- | --- | --- |
| Season of sample production | |  |  |  |
|  | Spring (%) | 34.8 |  |  |
|  | Summer (%) | 21.5 |  |  |
|  | Autumn (%) | 22.9 |  |  |
|  | Winter (%) | 20.9 |  |  |
|  |  |  |  |  |
| Time in the mail | |  |  |  |
|  | 1 day (%) | 24.7 |  |  |
|  | 2 days (%) | 35.5 |  |  |
|  | 3 days (%) | 18.1 |  |  |
|  | 4 days (%) | 15.9 |  |  |
|  | 5 days (%) | 5.8 |  |  |
|  |  |  |  |  |
| DNA isolation batch | |  |  |  |
|  | Batch 0 (%) | 91.4 |  |  |
|  | Batch 1 (%) | 8.6 |  |  |
|  |  |  |  |  |
| Sequencing batch | |  |  |  |
|  | Batch 0 (%) | 84.4 |  |  |
|  | Batch 1 (%) | 0.2 |  |  |
|  | Batch 2 (%) | 3.4 |  |  |
|  | Batch 3 (%) | 12 |  |  |
|  |  |  |  |  |
| Number of reads | |  | 20,196 (11,918) |  |
|  |  |  |  |  |
| Alpha diversities | |  |  |  |
|  | observed ASVs |  | 149.10 (49.2) |  |
|  | Shannon |  | 3.98 (0.42) |  |
|  | Inversed Simpson |  | 31.50 (14.18) |  |
|  |  |  |  |  |
| Number of taxa | |  |  |  |
|  | ASVs |  | 149.1 (49.2) | 1,578 |
|  | Species |  | 84.2 (20.1) | 305 |
|  | Genus |  | 61.1 (14.6) | 188 |
|  | Family |  | 25.5 (5.8) | 64 |
|  | Order |  | 16.1 (3.6) | 33 |
|  | Class |  | 8 (1.8) | 17 |
|  | Phylum |  | 5.2 (1.1) | 10 |

ASVs: amplicon sequence variants

**Supplementary Table 3. Descriptives child mental health measures**

|  | Mean (SD) | Range | N above clinical cutoff (%) |
| --- | --- | --- | --- |
| Overall psychiatric symptoms |  |  |  |
| Internalizing problems | 4.79 (4.90) | 0-37 | 120 (6.76) |
| Externalizing problems | 3.82 (4.61) | 0-38 | 47 (2.65) |
| Total problems | 17.51 (15.09) | 0-126 | 70 (3.95) |
| Specific domains of emotional and behavioral problems | | |  |
| Anxious/depressed | 2.19 (2.61) | 0-18 | 85 (4.79) |
| Withdrawn/depressed | 1.15 (1.66) | 0-11 | 128 (7.22) |
| Somatic complaints | 1.43 (1.87) | 0-14 | 121 (6.82) |
| Social problems | 1.67 (2.17) | 0-16 | 63 (3.55) |
| Thought problems | 1.64 (2.11) | 0-15 | 116 (6.54) |
| Attention problems | 3.24 (3.15) | 0-18 | 105 (5.92) |
| Rule-breaking behavior | 0.98 (1.39) | 0-10 | 38 (2.14) |
| Aggressive behavior | 2.84 (3.61) | 0-28 | 60 (3.38) |

CBCL: Child Behavior Checklist, the child mental health problems; SD: standard deviation. Clinical cutoff is a (age and sex standardized) T-score of 65 and higher.

**Supplementary Table 4. Associations between child mental health problems and overall gut microbiome composition (genus-level) in a subset of participants with time in mail of the sample 3 days or less**

|  | Microbiome richness | | |  | Shannon diversity | | |  | Inversed Simpson diversity | | |
| --- | --- | --- | --- | --- | --- | --- | --- | --- | --- | --- | --- |
|  | B | se | *p* |  | B | se | *p* |  | B | se | *p* |
| Overall psychiatric symptoms |  |  |  |  |  |  |  |  |  |  |  |
| Internalizing problems | -0.97 | 0.75 | .196 |  | -0.01 | 0.01 | .120 |  | -0.66 | 0.33 | .046 |
| Externalizing problems | 0.22 | 0.73 | .761 |  | 0.00 | 0.01 | .850 |  | 0.10 | 0.32 | .746 |
| Total problems | -0.37 | 0.51 | .460 |  | -0.01 | 0.01 | .284 |  | -0.25 | 0.22 | .260 |
| Specific domains of emotional and behavioral problems | | |  |  |  |  |  |  |  |  |  |
| Anxious/depressed | -1.67 | 0.92 | .069 |  | -0.02 | 0.01 | .063 |  | -0.92 | 0.40 | .022 |
| Withdrawn/depressed | -0.26 | 1.09 | .814 |  | -0.01 | 0.01 | .469 |  | -0.11 | 0.48 | .825 |
| Somatic complaints | -0.19 | 1.05 | .855 |  | 0.00 | 0.01 | .795 |  | -0.47 | 0.46 | .307 |
| Social problems | -0.29 | 1.00 | .774 |  | -0.02 | 0.01 | .182 |  | -0.65 | 0.44 | .140 |
| Thought problems | -0.85 | 1.01 | .400 |  | -0.02 | 0.01 | .201 |  | -0.55 | 0.45 | .218 |
| Attention problems | 0.29 | 0.88 | .743 |  | 0.00 | 0.01 | .719 |  | 0.20 | 0.39 | .610 |
| Rule-breaking behavior | 0.17 | 1.19 | .887 |  | -0.01 | 0.01 | .696 |  | -0.14 | 0.53 | .786 |
| Aggressive behavior | 0.08 | 0.80 | .917 |  | 0.00 | 0.01 | .779 |  | 0.13 | 0.35 | .714 |

Linear regression of child mental health problems and gut microbiome alpha diversities. Model: alpha diversity measure ~ sqrt(child mental health phenotype) + age + sex + BMI + self-reported use of antibiotics + maternal education + time in mail + season of stool production + DNA isolation batch + sequencing run batch + number of sequencing reads + first 10 genetic PCs. Values are pooled from 30 imputed datasets. Alpha: alpha diversity metric; sqrt: square root transformed; PCs: principal components; B: beta; se: standard error; p: p-value before correction.

**Supplementary Table 5. Associations between child mental health problems and overall gut microbiome composition: PERMANOVA (genus-level)**

| Child mental health | *F* | *p* | R^2^ |
| --- | --- | --- | --- |
| Broadband and total scales |  |  |  |
| Internalizing problems | 1.197 | .181 | 0.065% |
| Externalizing problems | 1.124 | .260 | 0.061% |
| Total problems | 1.184 | .193 | 0.064% |
| Empirical scales |  |  |  |
| Anxious/depressed | 1.020 | .407 | 0.055% |
| Withdrawn/depressed | 0.934 | .566 | 0.051% |
| Somatic complaints | 1.202 | .179 | 0.066% |
| Social problems | 0.866 | .689 | 0.047% |
| Thought problems | 0.974 | .487 | 0.053% |
| Attention problems | 0.957 | .519 | 0.052% |
| Rule-breaking behavior | 1.370 | .073 | 0.074% |
| Aggressive behavior | 0.874 | .675 | 0.047% |

PERMANOVA of child mental health problems and gut microbiome composition. Model: distance matrix ~ age + sex + BMI + self-reported use of antibiotics + maternal education + time in mail + season of stool production + DNA isolation batch + sequencing run batch + number of sequencing reads + first 10 genetic PCs + sqrt(child mental health problems). The distance matrix was generated from the filtered genus-level taxonomy table after centered log ratio transformation. Values are medians of 30 iterations that include different imputed datasets. Sqrt: square root; PCs: principal components; R^2^: percentage explained variance; *p*: *p*-value before correction
